# 

**Published:** 1885-07

**Authors:** 


### CONTENTS.

COMMUNICATIONS.

Oxy-phosphate .......... 341
Dental Economics .......... 342–345
Cannabis Indica .......... 347
Popular Dental Education .......... 347
Extracting Free .......... 352

PROCEEDINGS.

Mad River Dental Society .......... 354
National Association of Dental Examiners .......... 364
SALMAGUNDI .......... 365

EDITORIAL.

International Medical Congress .......... 370
Patriotic .......... 371
Pennsylvania State Dental Society .......... 371
An Unusual Case .......... 372
American Dental Association .......... 373
National Association of Dental Examiners .......... 373
Educational .......... 373
A Peculiar Case .......... 374
Hymenial .......... 375

